# Evaluation of Betanin on Key Enzymes Related to Obesity, Diabetes, Insulin Signaling Pathway, and Metabolic Disorders: In Vitro, Cellular, and In Silico Study

**DOI:** 10.3390/ph19060947

**Published:** 2026-06-16

**Authors:** Faiza I. A. Abdella, Dalal Alardan, Nawal S. Alshammari, Ahlam Abdulrahman Alrashdi, Mourad Jridi, Sarra Boudriga, Khaled Hamden

**Affiliations:** 1 Department of Chemistry, College of Science, University of Ha’il, Ha’il 81451, Saudi Arabia; 2 Department of Biology, College of Science, University of Ha’il, Ha’il 81451, Saudi Arabia; 3 Laboratory of Functional Physiology and Valorization of Bio-Resources (LR23ES08), Higher Institute of Biotechnology of Beja (ISBB), University of Jendouba, Beja 9000, Tunisia; 4 Laboratory of Heterocyclic Chemistry Natural Product and Reactivity (LR11ES39), Department of Chemistry, Faculty of Science of Monastir, University of Monastir, Monastir 5019, Tunisia; 5 Laboratory of Bioresources: Integrative Biology and Exploiting, Higher Institute of Biotechnology of Monastir, University of Monastir, Monastir 5000, Tunisia; 6 Higher School of Health Sciences and Technology of Sfax, Sfax University, Sfax 3000, Tunisia

**Keywords:** in silico, in vitro, betanin, digestive enzymes, metabolic regulation, diabetes

## Abstract

**Background/Objectives**: Betanin (Bet), a natural compound, exhibits potent antioxidant and metabolic regulatory properties, yet its effect on cellular glucose utilization remains unclear. This study investigated, for the first time, the impact of Bet on glucose consumption and the activation of key carbohydrate–catabolic pathways in human erythrocytes. **Methods**: In vitro assays were performed to evaluate enzyme inhibition and activation. Human erythrocytes were incubated with Bet to assess glucose consumption. Enzyme activities were measured spectrophotometrically, and molecular docking was used to analyze binding interactions. **Results**: Our results demonstrate that Bet inhibits digestive enzymes in a dose-dependent manner, with maximal inhibition at 90 µg/mL for pancreatic lipase and 70 µg/mL for α-amylase, showing IC_50_ values of 48.8 and 31.9 µg/mL, respectively, supported by strong binding affinities of −9.3 and −8.9 Kcal/mol. These interactions are stronger than those of orlistat (−6.9 Kcal/mol) and acarbose (−7.7 Kcal/mol). Bet also induced the activity of AMPK with an IC_50_ of 1.83 µg/mL and a BE of −7.90 Kcal/mol, compared to the specific AMPK activator A-769662, which had an IC_50_ of 1.29 µg/mL and a binding energy of −10.0 Kcal/mol. Consequently, Bet stimulated key glycolytic enzymes, reaching maximal activation (~62%) at 1.4 µg/mL for hexokinase (HK) and glucose-6-phosphate dehydrogenase (G6PD), and at 1.6 µg/mL for pyruvate kinase (PK), supported by binding energies of −7.2, −7.5, and −9.0 Kcal/mol and AC_50_ values of 0.87, 0.98, and 0.91 µg/mL, respectively. Moreover, Bet enhanced key Krebs cycle enzymes (IDH, SDH, MDH, LDH) in a dose-dependent manner, with AC_50_ values of 0.76, 0.80, 0.72, and 0.52 µg/mL and strong binding energies (−7.8, −7.8, and −8.4 Kcal/mol), reaching maximal activation near 1.4 µg/mL. Bet also increased glucose consumption by human erythrocytes. **Conclusions**: Bet enhances glucose utilization by inhibiting digestive enzymes and activating intracellular metabolic pathways, suggest potential metabolic regulatory effects.

## 1. Introduction

Obesity and type 2 diabetes (T2D) represent major global public health challenges due to their rising prevalence and severe impact on morbidity and mortality. In 2022, the World Health Organization (WHO) reported that one in eight people worldwide was living with obesity, and the prevalence of adult obesity having more than doubled since 1990 [1]. The World Obesity Federation estimated that over 1 billion people, including 880 million adults and 159 million children/adolescents, are obese, reflecting a rapid epidemiological transition from undernutrition to a double burden where underweight and overweight coexist [2]. According to WHO, 2.5 billion adults (43%) were overweight in 2022, and 890 million (16%) were obese, more than doubling since 1990 [3]. The health consequences are substantial: over 2.8 million deaths annually and 35.8 million disability-adjusted life years are attributed to overweight or obesity [4]. These alarming trends underscore the urgent need for effective prevention, early detection, and management strategies to mitigate obesity and its associated metabolic disorders worldwide. The global epidemic of obesity is strongly linked to the rising burden of type 2 diabetes (T2D). A global analysis reported that in 2021, around 506 million people were living with T2D, reflecting a nearly 290% increase since 1990, with body mass index being the leading risk factor contributing to diabetes-related deaths and disability-adjusted life years [5]. Another recent study confirmed that high BMI is the major contributor to the global burden of T2D, underscoring the importance of obesity as a modifiable risk factor [6]. The International Diabetes Federation projects that by 2025, approximately 11.1% of adults (1 in 9) aged 20–79 years will be living with diabetes, further highlighting the magnitude of the diabetes epidemic [7].

The health consequences of obesity and T2D are profound, affecting multiple organ systems and significantly reducing life expectancy [8,9]. Obesity is a well-established risk factor for hypertension, dyslipidemia, and atherosclerosis, which increase the risk of myocardial infarction and stroke, while T2D is associated with macrovascular complications, such as coronary heart disease, stroke, and peripheral arterial disease, as well as highly prevalent microvascular complications, including retinopathy, nephropathy, and neuropathy, which are linked to cognitive decline and depressive symptom [10]. Synthetic drugs, including metformin, acarbose, and orlistat, are widely used to manage obesity and T2D by reducing hepatic glucose production, improving insulin sensitivity, delaying carbohydrate absorption, and limiting dietary fat uptake; however, their long-term use is often associated with adverse effects such as gastrointestinal discomfort, vitamin deficiencies, lactic acidosis, and fat-soluble vitamin malabsorption [11,12]. Given the serious health consequences of diabetes and the limitations of synthetic drugs, there is growing interest in identifying safe natural bioactive compounds capable of managing diabetes, associated metabolic disorders, and related health disturbances, providing a sustainable strategy for preserving human health.

Natural bioactive compounds have gained considerable attention as potential alternatives because of their capacity to target multiple pathways simultaneously [13,14,15,16,17,18]. Unlike single-target synthetic drugs, these compounds often exhibit pleiotropic effects, including modulation of enzymatic activities, regulation of signaling pathways, and improvement of metabolic networks [19,20,21,22,23,24,25]. This multi-target potential allows natural compounds to exert more comprehensive control over glucose and lipid metabolism, potentially mitigating the progression of obesity and T2D. Furthermore, many bioactive molecules possess additional health-promoting properties, such as antioxidant activity, which protects against oxidative stress; anti-inflammatory effects, which reduce chronic low-grade inflammation; and immunomodulatory actions, which support immune homeostasis. These combined activities are particularly beneficial in metabolic disorders, where oxidative stress, chronic inflammation, and immune dysregulation play key roles in disease progression and complications.

Among natural compounds, betanin (Bet, C_24_H_26_N_2_O_13_),a water-soluble heterocyclic pigment responsible for the red coloration of *Opuntia stricta* [15] and related plants, has emerged as a promising candidate. Bet has been extensively studied for its pharmacological properties, demonstrating anticancer effects through modulation of apoptosis and cell cycle, anti-inflammatory effects via inhibition of pro-inflammatory mediators such as TNF-α and IL-6, and immunomodulatory effects by enhancing macrophage and lymphocyte function [15,26]. Additionally, Bet exhibits antioxidant properties, scavenging reactive oxygen species (ROS) and protecting cellular macromolecules from oxidative damage. These pleiotropic activities suggest that Bet may have the capacity to modulate multiple metabolic pathways simultaneously [15]. Its chemical structure, which includes heterocyclic rings and multiple hydroxyl groups, allows it to interact with enzymes and proteins through hydrogen bonding, hydrophobic interactions, and π–π stacking, influencing enzymatic activities and metabolic flux. Collectively, these characteristics indicate that Bet could serve as a multi-target natural agent with the potential to improve glucose and lipid homeostasis, reduce oxidative stress and inflammation, and support immune function, making it an attractive candidate for managing metabolic disorders such as obesity and T2D.

No previous studies have investigated the effect of Bet on the activity of key carbohydrate–catabolic enzymes in glycolysis and the Krebs cycle, either at the enzymatic, in silico, cellular, or in vivo level. In this context, the present study aimed, for the first time, to evaluate the effects of Bet on key enzymes involved in digestion, glycolysis, and the Krebs cycle. Specifically, we evaluated the inhibitory activity of Bet on digestive enzymes, including pancreatic lipase and α-amylase, as well as its activating effects on key enzymes of insulin signaling pathways as AMPK, glycolysis as HK, G6PD, and PK, and the Krebs cycle, namely SDH, MDH, IDH, and LDH. A multi-tiered approach was employed, combining vitro enzymatic assays, in silico molecular docking to identify binding sites and predict interaction strengths, and cellular assays using human cells under controlled incubation conditions.

## 2. Results

### 2.1. Bet and the Activity of Key Digestive Enzymes Related to Obesity and Type 2 Diabetes

Our study demonstrated that betanin (C_24_H_26_N_2_O_13_) exhibited a dose-dependent inhibitory effect, with maximum inhibition observed at 90 µg/mL and an IC_50_ value of 48.8 µg/mL, comparable to that of the standard inhibitor orlistat (IC_50_ = 49 µg/mL) (Ref.). Molecular docking analysis further supported these findings, revealing that Bet forms a strong and stable complex with lipase (BE = −9.3 Kcal/mol), indicating a higher binding affinity than orlistat (BE = −6.9 Kcal/mol). This enhanced interaction is attributed to multiple hydrogen bonds with key residues (TYR114, PHE215, SER152, HIS151, and THR115), along with π–π stacking with PRO180 and hydrophobic alkyl interactions involving ALA260 and HIS263, ensuring optimal ligand stabilization within the active site. In contrast, orlistat exhibited moderate binding affinity, stabilized by hydrogen bonds with HIS151, TYR114, and HIS263, electrostatic interaction with ASP79, and hydrophobic contacts with PHE77, ILE78, LEU264, and ALA259. Collectively, these findings suggest that Bet may act as a more potent lipase inhibitor than orlistat.

**Figure 1:**
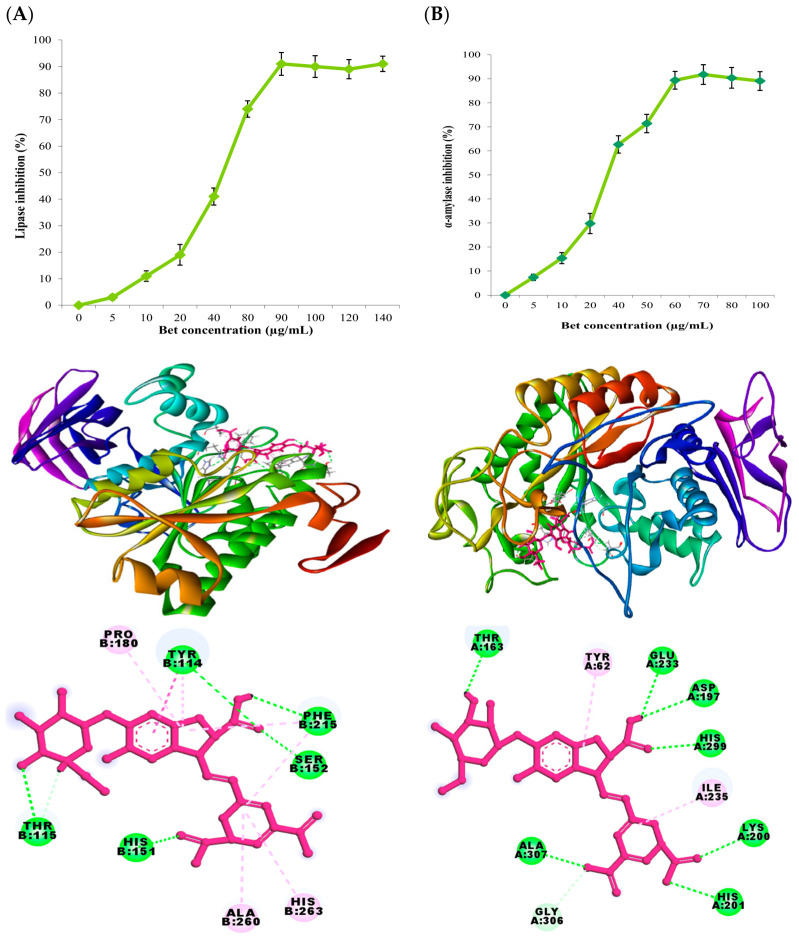
Inhibitory effects of Bet on pancreatic lipase (**A**) and α-amylase (**B**). Dose-dependent in vitro inhibition showed maximal activity at 90 µg/mL for lipase and 70 µg/mL for α-amylase. Molecular docking revealed strong interactions of Bet with key residues, including hydrogen bonds, π–π stacking, and alkyl contacts. These interactions confer durable and potent inhibition compared to metformin.

Regarding α-amylase, this enzyme is essential for carbohydrate digestion, catalyzing the breakdown of starch into oligosaccharides and glucose, thus playing a critical role in regulating postprandial glycemia. In the present study, Bet demonstrated strong inhibitory activity, supported by molecular docking results showing a high binding affinity (BE = −8.9 Kcal/mol). This strong interaction is mediated by multiple hydrogen bonds with key catalytic residues, including Asp300, Asp353, Asp356, Glu233, His305, and Thr163, which are essential for enzyme function. In comparison, acarbose exhibited a moderate binding affinity (BE = −7.7 Kcal/mol), lower than that of Bet, with stabilization mainly driven by hydrogen bonding and electrostatic interactions between negatively charged residues (Asp and Glu) and the hydroxyl groups of the ligand, along with a weak π–interaction with Trp59 that contributes to ligand orientation. These results indicate that Bet may provide a more stable and sustained inhibitory effect than acarbose, highlighting its potential as a natural antidiabetic agent (Figure 1; Table 1).

### 2.2. Effect of Bet on Key Enzymes Regulating the Insulin Signaling Pathway as AMPK

**Figure 2:**
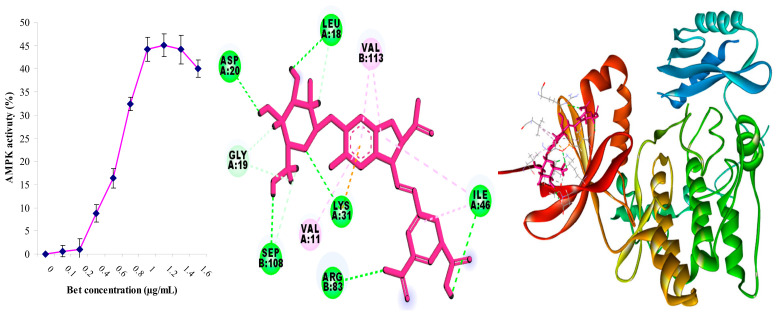
Effect of Bet on the activity of the key insulin-signaling enzyme AMPK. Results show that Bet activates insulin signaling, with maximal activation observed at 1 µg/mL, reaching 44.3% activation.

As shown in Figure 2, Bet induced a concentration-dependent activation of AMPK in the human liver-derived enzyme (SRP5003). At low concentrations (0.1–0.2 µg/mL), Bet produced only modest stimulation of AMPK activity. This activation increased progressively with rising concentrations, reaching 44.3% at 1 µg/mL and peaking at 45.1% at 1.2 µg/mL, indicating an optimal activation range. Beyond this concentration, a gradual decline in activity was observed, suggesting a bell-shaped dose–response curve. The IC_50_ value of Bet was determined to be 1.83 µg/mL, compared to 1.29 µg/mL for the specific AMPK activator A-769662, used as a positive control to validate the assay (Table 1).

Molecular docking analysis further supported these results, revealing that Bet interacts with AMPK through a diverse network of stabilizing interactions, with a moderate binding affinity (BE = −7.9 Kcal/mol). The binding is mainly stabilized by multiple hydrogen bonds involving key residues such as LYS29, LYS31, SER108, GLY19, and ARG83, which contribute to ligand anchoring within the binding pocket. In addition, hydrophobic interactions with residues including VAL11, VAL81, VAL113, LEU18, and ILE46, as well as a π–π stacking interaction with PHE90, further enhance ligand stabilization and proper orientation within the active site. In comparison, the reference activator A-769662 exhibited a stronger binding affinity (BE = −10 Kcal/mol), reflecting a more favorable and stable interaction due to its optimized structural complementarity and more extensive interaction network. Despite this, Bet’s ability to form multiple stabilizing interactions with key residues suggests that it can effectively modulate AMPK activity (Figure 2).

### 2.3. Bet and Key Glycolytic Enzymes

**Figure 3:**
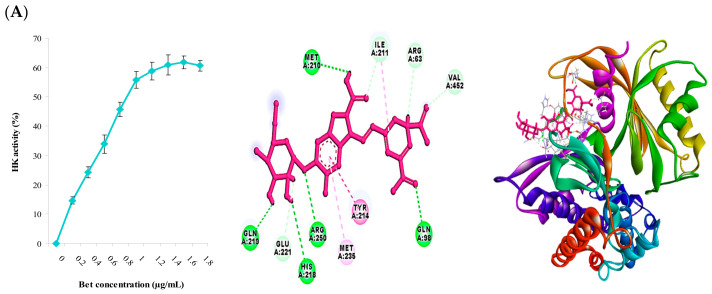

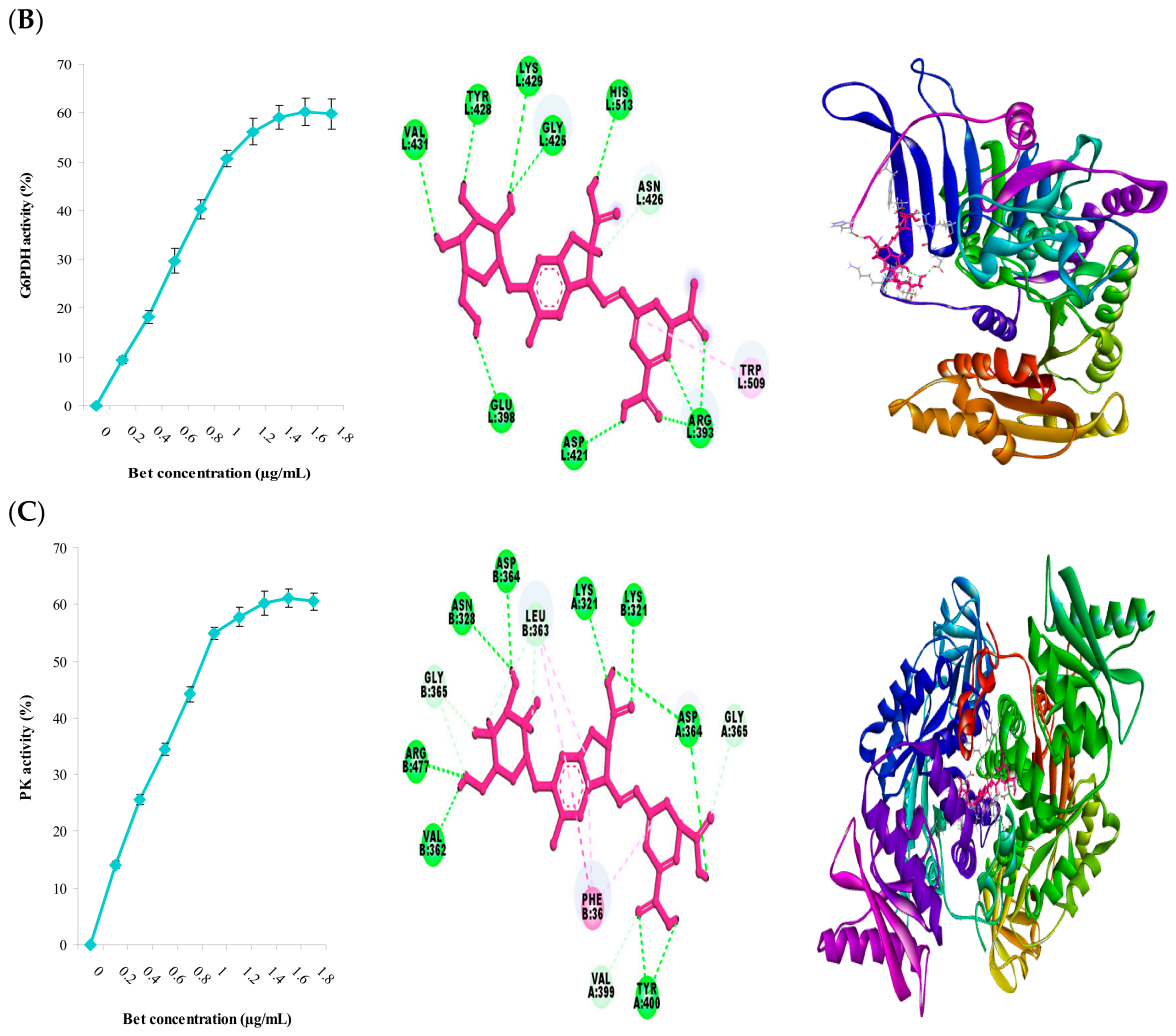
Activation of key glycolytic enzymes including HK (**A**), G6PD (**B**), and PK (**C**) by Bet. Bet enhanced enzyme activities in a dose-dependent manner, with AC_50_ values of 0.87 µg/mL (**A**), 0.98 µg/mL (**B**), and 0.91 µg/mL (**C**), respectively. Molecular docking analysis revealed multiple stabilizing interactions, including hydrogen bonds, C–H interactions, π–π stacking, and alkyl contacts, which contributed to strong binding affinity. In contrast, metformin exhibited weaker binding and fewer interaction types.

The results of this study indicate, for the first time, that Bet was shown to enhance the activity of key glycolytic enzymes, including HK, G6PD, and PK, in a dose-dependent manner. Maximal activating concentrations were 1.4 µg/mL for HK and G6PD and 1.6 µg/mL for PK, with maximal activation not exceeding 62%, suggesting physiologically controlled modulation without overactivation. These effects were further supported by half-maximal activating concentrations (AC_50_) of 0.87, 0.98, and 0.91 µg/mL for HK, G6PD, and PK, respectively. The potent activation of these enzymes is reinforced by strong binding interactions between Bet and the enzymes, with binding energies of −7.2, −7.5, and −9.0 Kcal/mol for HK, G6PD, and PK, respectively. Detailed analysis revealed diverse interactions: HK forms hydrogen bonds with MET210, GLN98, GLN219, ARG250, and HIS218, C–H interactions with ILE211, ARG63, VAL452, and GLU221, π–π stacking with TYR214, and alkyl interactions with MET235; G6PD engages via hydrogen bonds with VAL431, TYR428, LYS429, GLY425, HIS513, ARG393, ASP421, and GLU398, C–H interactions with ASN426, and an alkyl bond with THR509; PK forms hydrogen bonds with ASN328, ASP364, LYS321, TYR400, VAL362, and ARG477, C–H interactions with GLY365, LEU363, and VAL399, and π–π stacking with PHE36. In contrast, metformin exhibits weaker interactions with these enzymes, with higher binding energies and fewer types and numbers of contacts, highlighting Bet strong and stable activating effect on glycolytic enzymes and its ability to promote carbohydrate catabolism (Figure 3; Table 1).

### 2.4. Effect of Bet on the Activity of Key Carbohydrate Catabolic Enzymes in the Krebs Cycle

**Figure 4:**
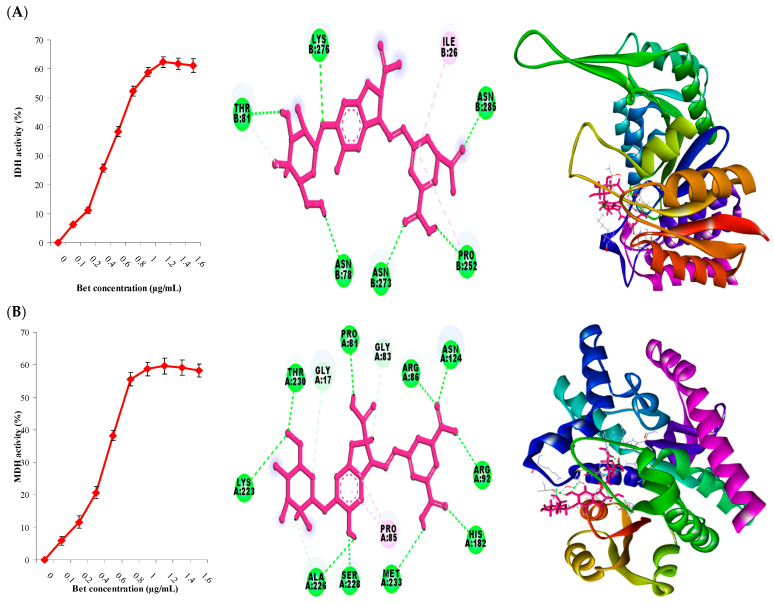

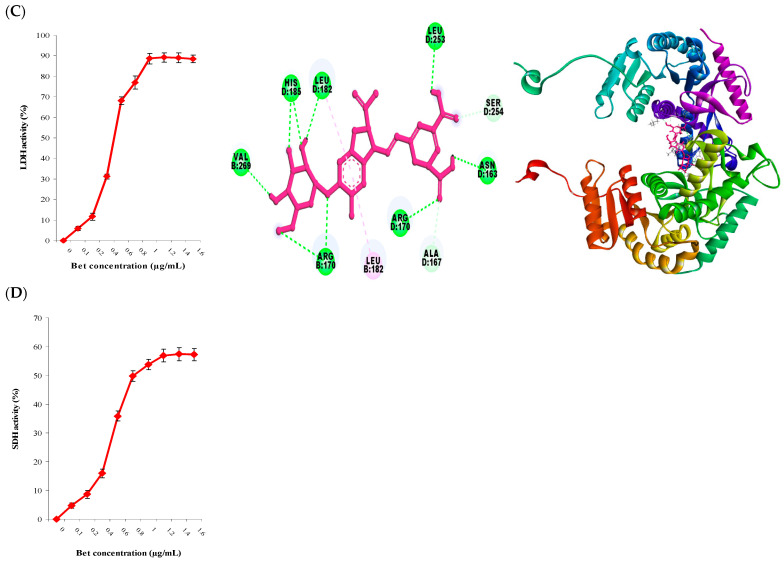
Activation of key Krebs cycle enzymes including IDH (**A**), SDH (**B**), MDH (**C**), and LDH (**D**) by betanin. Betanin enhanced enzyme activities in a dose-dependent manner, with AC_50_ values of 0.76 µg/mL (**A**), 0.80 µg/mL (**B**), 0.72 µg/mL (**C**), and 0.52 µg/mL (**D**), respectively. Molecular docking analysis revealed multiple stabilizing interactions for IDH, MDH, and LDH, including hydrogen bonds, C–H interactions, and alkyl contacts, contributing to strong binding affinity. The binding site of betanin on SDH could not be determined due to the lack of available structural information. In contrast, metformin exhibited weaker binding energies and fewer interactions, indicating lower activation potential.

In this study, Bet demonstrated a dose-dependent activating effect on key enzymes of the Krebs cycle, specifically IDH, SDH, MDH, and LDH. Maximal activating concentrations were approximately 1.4 µg/mL, highlighting Bet strong capacity to enhance carbohydrate metabolism. The half-maximal activating concentrations (AC_50_) were 0.76 µg/mL for IDH, 0.80 µg/mL for SDH, 0.72 µg/mL for MDH, and 0.52 µg/mL for LDH, whereas metformin exhibited fewer and weaker interactions with these enzymes. Molecular docking analyses revealed strong and diverse interactions: IDH formed hydrogen bonds with THR81, LYS276, ASN273, ASN285, PRO252, and an alkyl interaction with ILE26 (binding energy −7.8 Kcal/mol); MDH showed ten hydrogen bonds, two C–H interactions, and one alkyl interaction (−7.8 Kcal/mol); LDH exhibited hydrogen bonds with VAL269, HIS185, LEU182, GLU253, ASN163, ARG170, a C–H interaction with ALA167, and an alkyl interaction with LEU182 (−8.4 Kcal/mol). SDH activity could not be confirmed in silico due to limited structural information. In contrast, metformin displayed fewer interactions and weaker binding, highlighting Bet potent and stable activation of key Krebs cycle enzymes. These findings support Bet ability to enhance carbohydrate catabolism and its potential anti-diabetic effects (Figure 4; Table 1).

### 2.5. Effect of Bet on Glucose Consumption by Human Erythrocytes

**Figure 5:**
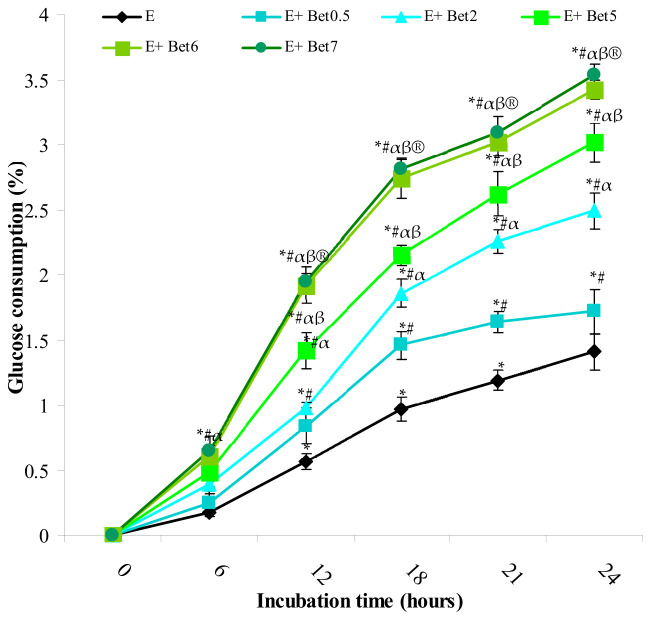
The results show that Bet induced a dose-dependent increase in glucose consumption by human erythrocytes, indicating enhanced peripheral glucose utilisation. Data are presented as the mean ± SD of three independent experiments. Statistical significance was determined using appropriate tests: * *p* ≤ 0.05 compared with untreated incubated erythrocytes; # *p* ≤ 0.05 compared with erythrocytes treated with Bet at 0.5 µg/mL; α *p* ≤ 0.05 compared with erythrocytes treated with Bet at 5 µg/mL; β *p* ≤ 0.05 compared with erythrocytes treated with Bet at 6 µg/mL; and ® *p* ≤ 0.05 compared with erythrocytes treated with Bet at 6 µg/mL.

Our study demonstrated that the incubation of human erythrocytes with Bet significantly enhanced glucose catabolism, as evidenced by a dose-dependent increase in glucose consumption. The maximal stimulatory concentration of Bet on glucose catabolism was observed at 6 µg/mL, beyond which glucose utilization reached a plateau. After 24 h of incubation, untreated erythrocytes consumed 1.41 µg/mL of glucose, whereas the presence of Bet at 0.5, 2, 5, 6, and 7 µg/mL increased glucose consumption to 1.72, 2.49, 3.02, 3.42, and 3.54 µg/mL, respectively. These results clearly demonstrate the potent stimulatory effect of Bet on glucose catabolism in human erythrocytes in vitro (Figure 5).

## 3. Discussion

Obesity and T2D are closely linked metabolic disorders, often associated with insulin resistance or a marked decrease in insulin activity. Insulin is a key hormone that regulates carbohydrate and lipid catabolism, ensuring the production of energy in the form of ATP, which is essential for optimal cellular function. Reduced insulin activity leads to impaired glucose and lipid metabolism, causing the accumulation of glucose and lipids in the blood and tissues. Our results are in accord with the study by Abbes et al., [27] which demonstrated a relationship between diabetes and a decline in carbohydrate catabolism at both cytosolic and mitochondrial levels. This accumulation of glucose in plasma, together with the decline in carbohydrate catabolism, leads to various toxic effects, initially manifested by a state of hyperglycemia. This metabolic overload triggers multiple deleterious processes, including systemic inflammation, oxidative stress, and dysfunction of vital organs such as the liver and kidneys. Typical metabolic complications include hyperglycemia and hyperlipidemia, which further increase cardiovascular risk and metabolic disturbances. These findings are in agreement with previous studies by AlRashidi et al. [17] and El-Sofany et al. [18], which demonstrated that diabetes mellitus is associated with a pronounced inflammatory state and increased oxidative stress. These alterations primarily affect the pancreas but also extend to other organs, including the liver, kidneys, and testes. Furthermore, the ATP deficit resulting from disrupted energy catabolism contributes to generalized fatigue and functional weakness.

Effective and sustainable therapeutic strategies aim to target both nutrient absorption and intracellular metabolism. Inhibition of digestive enzymes such as pancreatic lipase and α-amylase reduces intestinal uptake of lipids and carbohydrates, thereby limiting metabolic overload. In parallel, stimulation of key enzymes involved in glucose catabolism, including cytosolic glycolytic enzymes and mitochondrial Krebs cycle enzymes, enhances energy utilization and ATP production.

Thus, inhibition of intestinal lipase represents an important therapeutic strategy for obesity management. Although Orlistat is an efficient FDA-approved synthetic lipase inhibitor widely used for weight control, its clinical application is limited by multiple gastrointestinal adverse effects including steatorrhea, oily spotting, fecal urgency, flatulence with discharge, abdominal cramps, fat-soluble vitamin deficiencies and poor long-term tolerance. Consequently, the search for natural compounds with potent lipase inhibitory potential and additional metabolic benefits including antioxidant, anti-inflammatory, and anti-diabetic properties is gaining increasing interest as safer alternatives [12,28]. In this context, our results demonstrate that Bet effectively inhibits pancreatic lipase in a dose-dependent manner, with an IC_50_ value closely comparable to Orlistat, suggesting that this natural pigment may represent a promising multifunctional nutraceutical for obesity and associated metabolic complications. This inhibitory effect of Bet on pancreatic lipase may involve several potential mechanisms: (1) Bet has a highly charged polyphenolic structure, rich in hydroxyl and amino groups, capable of forming stable hydrogen bonds and electrostatic interactions with amino acids in the lipase active site, thereby reducing substrate (triglyceride) access to the catalytic cavity; (2) Bet may act as a competitive or partially competitive inhibitor by structurally mimicking lipid ester groups, decreasing enzyme–substrate affinity; (3) its amphiphilic nature facilitates insertion at the water–lipid interface, a critical region for lipase interfacial activation, thus disrupting the conformational shift to the “open” active form; and (4) its antioxidant properties can modulate the lipid microenvironment by limiting oxidation, which further reduces lipase catalytic efficiency. In fact, the possible strong affinity of Bet for pancreatic lipase is supported by its binding energy of −9.3 Kcal/mol, indicating a highly stable interaction within the enzyme’s catalytic pocket [29], specifically around the catalytic triad Ser152–Asp176–His263. Docking analysis shows that Bet forms multiple hydrogen bonds with key residues such as Gln219, Glu221, His218, and Arg250, firmly anchoring the molecule in the active site. Its numerous hydroxyl (–OH) and amino (–NH_2_) groups also engage in electrostatic interactions with positively charged residues including Arg63 and Arg250, while the aromatic backbone of Bet participates in van der Waals contacts with hydrophobic residues such as Met210, Ile211, Tyr214, and Val452. In addition, the amphiphilic nature of Bet facilitates its partial insertion into the hydrophobic catalytic tunnel, physically hindering access of triglyceride substrates to the catalytic serine. Together, these cooperative interactions explain the strong inhibitory effect of Bet on pancreatic lipase activity in vitro, consistent with its IC_50_ value, which is similar to that of orlistat. Our results are consistent with those reported by El-Sofany et al., [15] who demonstrated that betanin extracted from *Opuntia stricta* var. dillenii inhibits pancreatic lipase in a dose-dependent manner in obese rat models. In addition, Pino et al. [30] showed that *Opuntia stricta* extract, in which betanin is the major component, inhibits pancreatic lipase with an IC_50_ value of 33.54 µg/mL.

The prevalence of diabetes, particularly T2D, is rising alarmingly worldwide, affecting over 900 million people and leading to severe metabolic, cardiovascular, inflammatory, and immune complications. Among the factors contributing to postprandial hyperglycemia, α-amylase, a salivary and intestinal enzyme, plays a central role by catalyzing the breakdown of dietary starches into oligosaccharides and maltose, which are subsequently absorbed as glucose. Excessive α-amylase activity promotes rapid glucose absorption, exacerbating hyperglycemia and insulin resistance, ultimately leading to progressive β-cell exhaustion and impaired glycemic control. Inhibition of α-amylase, as achieved by acarbose, slows starch digestion and glucose absorption, reducing postprandial glycemic spikes; however, such drugs often produce side effects that limit their clinical use. Natural compounds with α-amylase inhibitory activity offer a promising alternative for diabetes prevention and management, with additional benefits including anti-inflammatory, immunomodulatory, and organ-protective effects. In this study, Bet was shown to inhibit α-amylase in a dose-dependent manner, with a maximal inhibitory concentration of 70 µg/mL, indicating saturation of the enzyme’s active sites, and an IC_50_ of 31.9 µg/mL, closely comparable to acarbose (IC_50_ = 27.4 µg/mL). The inhibition of α-amylase by Bet can be explained through multiple molecular mechanisms. Firstly, the polyphenolic structure of Bet, along with its multiple hydroxyl (-OH) and amino (-NH_2_) groups, promotes the formation of hydrogen bonds with polar and charged residues in the enzyme’s active site, thereby reducing substrate access to the catalytic centers. Secondly, the partial charges and polarity of the molecule enable electrostatic interactions with charged amino acids surrounding the active site, while van der Waals forces stabilize a non-catalytic enzyme inhibitor complex, preventing proper substrate orientation. Moreover, the amphiphilic nature of Bet allows partial insertion at the water–substrate interface, disrupting the dynamics of the active site and limiting the flexibility required for catalysis. The docking analysis reveals that Bet binds strongly to the active site of human pancreatic α-amylase (PDB ID 5E0F) [31], with a binding energy of approximately −8.9 Kcal/mol. This high affinity correlates with the observed IC_50_ of 31.9 µg/mL. In the predicted complex, Bet forms multiple hydrogen bonds with key residues lining the catalytic cleft, while its polarized and charged groups engage in electrostatic interactions with amino acids near the active site. Furthermore, hydrophobic regions of the ligand participate in van der Waals contacts with residues within the substrate-binding groove, enhancing complex stability. The amphiphilic nature of Bet likely allows partial insertion into the enzyme’s interface region, thereby disrupting substrate access and catalytic turnover. No previous study has specifically investigated the strong interaction between α-amylase and betanin using molecular docking. However, the inhibitory effect of betanin against this key diabetes-related enzyme, α-amylase, has already been reported. For instance, El-Sofany et al. [15] demonstrated a dose-dependent suppressive effect of betanin on intestinal α-amylase activity, which was associated with an antihyperglycemic effect in HFFD rat models.

Induction of AMPK represents a promising strategy for the treatment of insulin resistance and associated metabolic disorders, as its activation enhances glucose uptake, stimulates glycolytic flux, and modulates key enzymes involved in energy homeostasis [32,33,34]. In this study, our results demonstrate that Bet induces AMPK activity in a dose-dependent manner, with the most effective concentration being 1 µg/mL, producing 44% enzyme activation. At higher concentrations, a slight and progressive decline in AMPK activity was observed, which may be attributed to reaction medium saturation and osmotic pressure changes potentially affecting the structural integrity of the enzyme active site. This strong activation of AMPK by Bet is likely due to its stable and high-affinity interaction with the enzyme, as indicated by low binding energy and multiple types of molecular interactions. These interactions promote a strong and sustained binding, resulting in more efficient enzyme activation compared to the specific AMPK activator. No previous in vitro or in silico study has specifically investigated the direct inhibitory or interactive effect of betanin on AMPK. However, previous studies have shown that Bet improves insulin signaling pathways and insulin sensitivity, as demonstrated by oral glucose tolerance tests [35]. In addition, Bet has been reported to induce AMPK gene expression in diabetic rat models [35]. Moreover, our findings are consistent with the molecular data reported by Abedimanesh et al. [35], who demonstrated that betanin intake in diabetic rats induces the gene expression of key proteins involved in the regulation of energy metabolism, insulin sensitivity, and protection of pancreatic β-cells. Specifically, betanin activates AMPK and SIRT1, two central regulators of metabolic homeostasis, while simultaneously suppressing NF-κB, a pro-inflammatory transcription factor whose chronic activation in diabetes leads to excessive production of inflammatory cytokines and contributes to insulin resistance. This activation of the AMPK–SIRT1 axis, which is known to enhance the transcriptional activity of PGC-1α and promote UCP1 expression, leads to increased thermogenesis and energy expenditure, while simultaneously stimulating lipolysis and glycolysis. Collectively, these metabolic effects contribute to the prevention of hyperglycaemia, in agreement with the findings reported by Lee et al. [36]. Simultaneously, activation of this signalling network, notably through nuclear factor erythroid-2-related factor 2, inhibits NF-κB-mediated inflammatory responses, thereby reducing the expression of pro-inflammatory cytokines that interfere with insulin signalling [37].

Induction of key glycolytic enzymes represents an important strategy for managing diabetes [38,39], as these enzymes catalyze the conversion of glucose into pyruvate in the cytoplasm, thereby generating cellular energy. In this study, we demonstrated for the first time that Bet appears to effectively stimulate key glucose-catabolic enzymes, potentially promoting glucose utilization and energy production. However, these findings require further confirmation in vitro and additional in vivo and clinical studies. Specifically, Bet appears to enhance the activities of HK, G6PD, and PK in a dose-dependent manner. The maximal activating concentrations were 1.4 µg/mL for HK and G6PD, and 1.6 µg/mL for PK, with overall activation not exceeding 62%, suggesting a controlled physiological modulation. The half-maximal activating concentrations (AC_50_) were 0.87 µg/mL for HK, 0.98 µg/mL for G6PD, and 0.91 µg/mL for PK, indicating a potent stimulatory effect of Bet that warrants further confirmation in vitro and in vivo, as well as in clinical studies. These findings suggest that Bet may promote glucose catabolism, which, following ingestion of this pigment, could lead to enhanced ATP production and contribute to a reduction in plasma glucose levels in diabetic patients. Bet demonstrated a strong and stable interaction with key glycolytic enzymes, including HK [40,41], G6PD [42], and PK [43]. Molecular docking analyses revealed that Bet binds to these enzymes with high affinity, exhibiting binding energies of −7.2, −7.5, and −9.0 Kcal/mol for HK, G6PD, and PK, respectively. The ligand–enzyme complexes are stabilized through multiple types of interactions, including hydrogen bonds (e.g., MET210, GLN98, ARG250 for HK; VAL431, TYR428, LYS429 for G6PD; ASN328, ASP364, TYR400 for PK), C–H interactions, π–π stacking (TYR214 in HK, PHE36 in PK), and alkyl contacts (MET235 in HK, LEU182 in PK), which together confer strong, durable, and physiologically relevant binding [40,41]. These diverse interactions allow Bet to effectively enhance the catalytic activity of glycolytic enzymes in a dose-dependent manner, promoting carbohydrate catabolism, ATP production, and energy supply for cellular functions. In contrast, metformin exhibits significantly weaker interactions, forming primarily hydrogen bonds with fewer residues (e.g., PHE77 in HK, ASN163 in G6PD, ASP364 in PK) and lacking stabilizing π–π or alkyl contacts, resulting in lower binding affinity (−4.6, −4.6, and −5.0 Kcal/mol) and reduced enzyme activation [42]. Consequently, Bet multi-modal binding profile translates into superior bioactivity, providing stronger and more sustained stimulation of glycolytic flux compared to metformin, highlighting its potential as a natural anti-diabetic agent capable of enhancing peripheral glucose utilization and improving metabolic control [40,41,42,43]. No previous study has reported a direct activating effect of betanin on these two key cytosolic enzymes involved in glucose catabolism. However, betanin has been shown to activate insulin signaling pathways through AMPK induction, which in turn promotes glucose catabolism. This process begins at the cytosolic level, where the activation of AMPK contributes to the upregulation of key glycolytic enzymes such as HK and G6PD, thereby enhancing glucose utilization. Our results are in agreement with the study by Dhananjayan et al. [44], who reported that administration of Bet at a dose of 20 mg/kg body weight in diabetic rats significantly enhanced carbohydrate catabolism, particularly glycolysis, as evidenced by the increased hepatic activity of HK and PK.

Activation of the Krebs cycle represents a promising therapeutic strategy for improving glucose homeostasis in diabetes [45,46,47]. In this context, Bet appears to exhibit a strong activating affinity toward key enzymes involved in carbohydrate catabolism. The half-maximal activating concentrations (AC_50_) were 0.76 µg/mL for IDH, 0.72 µg/mL for MDH, and 0.52 µg/mL for LDH, with corresponding binding energies of −7.8, −7.8, and −8.4 Kcal/mol, respectively, suggesting a potent stimulatory effect that warrants further confirmation in vitro, in vivo, and in clinical studies. Detailed molecular docking analyses revealed diverse interactions that stabilize the enzyme-ligand complexes: IDH formed hydrogen bonds with THR81, LYS276, ASN273, ASN285, PRO252, and an alkyl interaction with ILE26; MDH exhibited ten hydrogen bonds, two C–H interactions, and one alkyl interaction; LDH formed hydrogen bonds with VAL269, HIS185, LEU182, GLU253, ASN163, ARG170, a C–H interaction with ALA167, and an alkyl interaction with LEU182. In contrast, metformin displayed weaker binding energies (−4.6 Kcal/mol across these enzymes) and fewer types of interactions, primarily hydrogen bonds, resulting in lower affinity and less sustained enzyme activation. The exact binding site of Bet on SDH could not be determined due to the lack of structural information. These findings suggest that Bet potent and multi-modal activation of key Krebs cycle enzymes may enhance carbohydrate catabolism and energy production, supporting its potential use as a natural anti-diabetic agent. Although no previous study has investigated the effect of betanin on key mitochondrial enzymes involved in glucose catabolism, highlighting the originality of the present work, the activation of insulin signaling by betanin, as reported in previous studies [36,44], may help explain its stimulatory effect on glucose catabolism. Our results are in agreement with the study by Lee et al., [36] who demonstrated that Bet extracts modulate cellular energy metabolism through activation of AMPK signaling, leading to enhanced glycolytic flux and improved mitochondrial function. These effects are associated with increased expression of key metabolic regulators such as PGC-1α and enhanced activity of mitochondrial pathways involved in ATP production and the Krebs cycle function.

Although the activity of digestive enzymes such as α-amylase was assessed using human enzymes, other enzymes were derived from non-human sources: pancreatic lipase from porcine pancreas, HK, G6PD, and PK from rabbit muscle, IDH from *Escherichia coli*, and SDH from porcine heart. These enzymes are widely recognized for their high structural and sequence similarity, particularly in the active site residues, which supports the reliability of the observed interactions and enzymatic results [48,49,50]. Nevertheless, this study has certain limitations

▪ Confirmation studies using fully human enzymes are necessary to human application.
▪ investigations in diabetic animal models and clinical studies in human diabetic subjects are required to validate the antidiabetic potential of Bet.
▪ Gastric stability, bioavailability, and pharmacokinetics of Bet to ensure its efficacy and applicability in vivo.

## 4. Materials and Methods

### 4.1. Chemicals and Reagents

All chemicals, reagents, substrates, and enzymes used in this study were purchased from Sigma-Aldrich (Merck, St. Louis, MO, USA) and used without further purification. Enzymes included pancreatic lipase (L0382), α-amylase (A6255), hexokinase (H8629), glucose-6-phosphate dehydrogenase (G6378), pyruvate kinase (P9136), isocitrate dehydrogenase (I2002), succinate dehydrogenase (S8471), malate dehydrogenase (M2383), and AMP-activated protein kinase (AMPK; SRP5003). The substrates and co-factors used in enzymatic assays included p-nitrophenyl palmitate, 2-chloro-4-nitrophenyl maltotrioside, glucose, ATP, ADP, NADH, NAD^+^/NADP^+^, glucose-6-phosphate, phosphoenolpyruvate, isocitrate, succinate, oxaloacetate, and 2,6-dichlorophenolindophenol (DCPIP). All buffers and additional reagents were of analytical grade, and solutions were prepared using deionized water. Statistical analysis was performed using StatView software, version 5.0 (SAS Institute Inc., Cary, NC, USA).

### 4.2. Extraction, Isolation, and Characterization of Bet from Opuntia stricta Var. Dillenii

The extraction of Bet from ripe and intact fruits of *Opuntia stricta* var. dillenii was performed according to the procedure described by El-Sofany et al., [15], under the supervision of Ali Mbarek, Agronomy Engineer. The plant was assigned a Voucher specimen number, OS1 at the herbarium of the Faculty of Sciences, Sfax University, Tunisia. The fruits (500 g) were washed and blended to obtain fresh juice, while the cladodes were peeled to remove the outer skin. For the extraction, 200 mL of juice was mixed with 400 mL of 1% HCl-acidified ethanol (*v*/*v*) and incubated at 4 °C for 2 h under gentle agitation (100 rpm) to preserve pigmentation, then filtered and centrifuged at 6000× *g* for 15 min at 4 °C. The supernatant was concentrated under vacuum at 35 °C to approximately 50 mL and deproteinized water, by adding 100 mL of cold ethanol (−20 °C) followed by centrifugation at 6000× *g* for 10 min. The extract was subsequently purified in two steps: first by solid-phase extraction (SPE) on a C18 cartridge pre-equilibrated with acidified water (0.1% HCl) and eluted with a methanol/water gradient (20–80%) to recover the red fractions, and then by column chromatography on silica gel (60–120 mesh) using a chloroform/methanol/acetic acid gradient (70:30:1 *v*/*v*/*v*). The colored fractions were combined, evaporated under vacuum, and lyophilized to obtain crude Bet, whose purity was verified by TLC (silica, ethyl acetate/methanol 9:1) and HPLC-UV (C18, water 0.1% formic acid/acetonitrile gradient, λmax ≈ 535 nm). Structural characterization was performed using UV-Vis spectroscopy, mass spectrometry (ESI-MS), and FT-IR.

### 4.3. Bet and Key Signalisation Insulin Pathway as AMPK Related to OB/T2D

AMPK activity was measured in vitro using a spectrophotometric coupled-enzyme assay. Human AMPK enzyme (SRP5003, Sigma, 0.05 µg/µL) was prepared in an assay buffer containing 50 mM HEPES, 10 mM MgCl_2_, 0.2 mM AMP, 0.5 mM ATP, 1 mM phosphoenolpyruvate, 0.15 mM NADH, 0.2 mg/mL pyruvate kinase, and 0.2 mg/mL lactate dehydrogenase. The enzyme was incubated at 37 °C for 5 min to allow equilibration with the reaction mixture. The reaction was then initiated by adding the AMPK-specific peptide substrate SAMS peptide (Sigma, S4878, 1 mM), and the decrease in NADH absorbance at 340 nm was continuously monitored to determine AMPK activity, expressed as nmol NADH oxidized per minute per mg protein [51]. All measurements were performed in triplicate. The assay was conducted in parallel with the specific AMPK activator A-769662 to validate enzyme responsiveness.

### 4.4. Bet and Key Digestive Enzymes Related to OB/T2D

The digestive enzymes evaluated were pancreatic lipase and salivary and pancreatic α-amylase, both obtained from commercial sources (L0382, Sigma-Aldrich, Merck, St. Louis, MO, USA). Pancreatic lipase (0.5 U/mL) catalyzes the hydrolysis of triglycerides into free fatty acids and monoglycerides. Its activity was measured using the chromogenic substrate p-nitrophenyl palmitate (0.5 mM) in 50 mM Tris-HCl buffer, pH 8.0, at 37 °C. The release of p-nitrophenol was monitored at 405 nm every 5 min for 30 min [52]. α-amylase (1 U/mL) hydrolyzes starch into dextrins and maltose, measured using 2-chloro-4-nitrophenyl maltotrioside (0.5 mM), absorbance at 405 nm [53]. Enzyme (A6255, Sigma-Aldrich) were pre-incubated with BET at 10, 20, 40, 60, 80, 160, 320, 640, and 1280 µg/mL for 30 min at 37 °C. A dose-dependent inhibition was observed, indicating that BET effectively reduces lipid and carbohydrate hydrolysis.

### 4.5. Bet and Key Cytosolic Glucose Catabolic Enzymes Related to OB/T2D

The in vitro activity of hexokinase (HK) was measured using Hexokinase from baker’s yeast (Saccharomyces cerevisiae, Sigma-Aldrich, Ref. H8629), dissolved in 50 mM Tris-HCl buffer, pH 7.4, containing 1 mM MgCl_2_, 1 mM EDTA, and 1 mM DTT to maintain enzyme stability. The reaction mixture (final volume 200 µL) contained 50 µL of enzyme solution (5 U/mL), 50 µL of 10 mM glucose (final 2.5 mM), 50 µL of 10 mM ATP (final 2.5 mM), 25 µL of 1 mM NADP^+^, and 25 µL of glucose-6-phosphate dehydrogenase (G6PDH, 1 U/mL) as a coupling enzyme to monitor NADPH formation. Reactions were incubated at 37 °C, and NADPH production was followed spectrophotometrically at 340 nm every 30–60 s for 3–5 min. Hexokinase activity was calculated based on the linear rate of NADPH formation and expressed as µmol NADPH produced per minute.

The in vitro activity of G6PDH was determined using Glucose-6-Phosphate Dehydrogenase from baker’s yeast (Saccharomyces cerevisiae, Sigma-Aldrich, Ref. G6378), dissolved in 50 mM Tris-HCl buffer, pH 7.4, containing 1 mM EDTA and 1 mM DTT. The reaction mixture (final volume 200 µL) contained 50 µL of enzyme solution (2 U/mL), 100 µL of Tris-HCl buffer with 1 mM MgCl_2_, 25 µL of 2 mM NADP^+^ (final 0.25 mM), and 25 µL of 2 mM glucose-6-phosphate (G6P, final 0.25 mM), while control reactions omitted G6P. Reactions were incubated at 37 °C, and NADPH formation was monitored at 340 nm every 30–60 s for 3–5 min. G6PDH activity was calculated from the linear rate of NADPH formation.

The in vitro activity of pyruvate kinase (PK) was measured using Pyruvate Kinase from rabbit muscle (Sigma-Aldrich, Ref. P9136), dissolved in 50 mM Tris-HCl buffer, pH 7.4, containing 1 mM MgCl_2_, 1 mM EDTA, and 1 mM DTT. The reaction mixture (final volume 200 µL) consisted of 50 µL of enzyme solution (5 U/mL), 50 µL of 2 mM phosphoenolpyruvate (PEP), 50 µL of 2 mM ADP, 25 µL of 1 mM NADH, and 25 µL of lactate dehydrogenase (LDH, 1 U/mL) as a coupling enzyme. Reactions were incubated at 37 °C, and NADH consumption was monitored at 340 nm every 30–60 s for 3–5 min. PK activity was calculated from the linear decrease in NADH absorbance and expressed as µmol NADH oxidized per minute.

### 4.6. Bet and Key Mitochondrial Glucose Catabolic Enzymes Related to OB/T2D

The in vitro activity of isocitrate dehydrogenase (IDH) was measured using IDH from Escherichia coli (Sigma-Aldrich, Ref. I2002), dissolved in 50 mM Tris-HCl buffer, pH 7.4, containing 1 mM MgCl_2_ and 1 mM DTT. The reaction mixture (final volume 200 µL) contained 50 µL of enzyme solution (5 U/mL), 50 µL of 2 mM NADP^+^, 50 µL of 2 mM isocitrate, and 50 µL of Tris-HCl buffer. Reactions were incubated at 37 °C, and NADPH formation was monitored spectrophotometrically at 340 nm every 30–60 s for 3–5 min. IDH activity was calculated from the linear increase in absorbance and expressed as µmol NADPH produced per minute.

The in vitro activity of succinate dehydrogenase (SDH) was measured using SDH from bovine heart (Sigma-Aldrich, Ref. S8471), dissolved in 50 mM phosphate buffer, pH 7.4, containing 1 mM EDTA. The reaction mixture (final volume 200 µL) contained 50 µL of enzyme solution (5 U/mL), 50 µL of 5 mM succinate, 50 µL of 0.5 mM 2,6-dichlorophenolindophenol (DCPIP) as an electron acceptor, and 50 µL of phosphate buffer. Reactions were incubated at 37 °C, and the reduction of DCPIP was monitored at 600 nm every 30–60 s for 3–5 min. SDH activity was calculated from the linear decrease in absorbance and expressed as µmol DCPIP reduced per minute.

The in vitro activity of malate dehydrogenase (MDH) was measured using MDH from porcine heart (Sigma-Aldrich, Ref. M2383), dissolved in 50 mM Tris-HCl buffer, pH 7.4, containing 1 mM EDTA and 1 mM DTT. The reaction mixture (final volume 200 µL) contained 50 µL of enzyme solution (5 U/mL), 50 µL of 2 mM NADH, 50 µL of 2 mM oxaloacetate, and 50 µL of Tris-HCl buffer. Reactions were incubated at 37 °C, and NADH consumption was monitored at 340 nm every 30–60 s for 3–5 min. MDH activity was calculated from the linear decrease in NADH absorbance and expressed as µmol NADH oxidized per minute.

### 4.7. Molecular Docking Procedure

The three-dimensional Bet was generated and energy-minimized using ACD 3D Viewer software to ensure a stable conformation for docking using AutoDock Vina 1.1.2 (The Scripps Research Institute, La Jolla, CA, USA). The crystal structures of the target enzymes were retrieved from the Protein Data Bank, including hexokinase (HK; PDB ID: 8XFD) [40,41], glucose-6-phosphate dehydrogenase (G6PD; PDB ID: 6E07) [42], pyruvate kinase (PK; PDB ID: 8XFD) [54], isocitrate dehydrogenase (IDH; PDB ID: 5GRF) [55], succinate dehydrogenase (SDH; PDB ID: 2H88) [56], malate dehydrogenase (MDH; PDB ID: 2DFD) [57], lactate dehydrogenase (LDH; PDB ID: 1LLD) [58], pancreatic lipase (PDB ID: 1LPB) [29], α-amylase (PDB ID: 5E0F) [59], and AMPK (PDB id = 4CFE) [60]. Prior to docking, all water molecules and co-crystallized ligands were removed, and polar hydrogens and Kollman charges were added to optimize the protein structures. Active site docking was performed using AutoDock Vina 1.1.2 [61], targeting the catalytic or substrate-binding pockets of each enzyme to predict binding modes, interaction energies, and key residues involved in ligand recognition. The docking results were analyzed in detail to identify hydrogen bonding, hydrophobic contacts, π–π stacking, and other non-covalent interactions between Bet and the enzymes. Interactions were visualized using BIOVIA Discovery Studio Visualizer, allowing structural interpretation of potential inhibitory or activating effects of Bet at the molecular level, thereby linking in silico predictions to observed enzymatic and cellular activities.

### 4.8. In Vitro Effect of Bet on Glucose Consumption by Human Erythrocytes

Fresh human venous blood was collected in EDTA-coated tubes and centrifuged at 1800 rpm for 10 min at 4 °C to remove plasma and the buffy coat. The erythrocyte pellet was washed three times with phosphate-buffered saline (PBS, 0.01 M, pH 7.4) and resuspended to a hematocrit of 10%. The culture medium consisted of PBS supplemented with 10 mmol/L glucose as a substrate for glycolysis and penicillin G (300 mg/L) plus streptomycin (500 mg/L) to prevent microbial contamination. Hemolysis was verified spectrophotometrically at 540 nm and remained below 1% in all preparations [62]. Aliquots of this erythrocyte suspension were pre-incubated with different concentrations of Bet (0.5–10 µg/mL) for 4 h at 37 °C in a shaking water bath, after which glucose consumption was monitored over 20 h under gentle agitation.

Aliquots of the erythrocyte suspension were distributed into sterile Erlenmeyer flasks and pre-incubated with different concentrations of isolated Bet at 0.5, 1, 2, 5, and 10 µg/mL for 4 h at 37 °C in a shaking water bath. After pre-incubation, glucose was added to each flask to reach a final concentration of 10 mmol/L, representing a physiologically relevant hyperglycemic condition. The suspensions were then incubated for an additional 24 h at 37 °C under gentle agitation to allow sufficient time for glucose uptake and metabolism by erythrocytes. Control groups included erythrocytes incubated without glucose (negative control) and erythrocytes incubated with glucose alone (positive control). This design allowed assessment of the effect of Bet on erythrocyte glucose consumption and metabolic activity under standardized in vitro conditions. During incubation, samples were collected every 6 h from each suspension, centrifuged at 1500× *g* for 10 min, and the glucose concentration in the supernatant was measured using the glucose oxidase–peroxidase (GOD-POD) colorimetric kit (Biolabo, ref 87409). Absorbance was recorded at 505 nm, and glucose consumption was calculated as the percentage decrease relative to the initial concentration.

During the experimental period, erythrocyte viability was monitored. After incubation, samples were centrifuged and the supernatant was analyzed for free hemoglobin by measuring absorbance at 540 nm (Biolabo kit, ref. 3502200), to assess potential hemolysis and, indirectly, membrane integrity. In addition, microscopic observation of cell morphology was performed to confirm the preservation of normal erythrocyte structure. Throughout the experiment, no significant hemolysis or detectable loss of cell viability was observed.

### 4.9. Statistical Analysis

Data from the hot plate test are presented as mean ± standard error of the mean (SEM). Each group included four repetitions. Statistical analysis was performed using StatView 5 software (SAS Institute Inc., USA). Differences between groups were assessed by one-way analysis of variance (ANOVA). When the overall ANOVA was significant (*p* < 0.05), multiple comparisons were conducted using the Tukey–Kramer post hoc test to identify groups with statistically significant differences. The threshold for significance was set at *p* < 0.05.

## 5. Conclusions

Bet appears to exhibit promising antidiabetic potential by modulating multiple key metabolic enzymes. It inhibits digestive enzymes such as pancreatic lipase and α-amylase, potentially reducing nutrient absorption. Bet activates AMPK, which in turn may stimulate glycolytic (HK, G6PD, PK) and Krebs cycle enzymes (IDH, MDH, LDH), thereby potentially enhancing carbohydrate catabolism, ATP production, and glucose utilization. Molecular docking analyses indicate that Bet forms stable, multi-modal interactions, supporting its binding affinity and durability. These findings highlight the possible multi-target therapeutic actions of Bet, which may offer advantages over conventional single-target antidiabetic drugs, such as acarbose, through complementary mechanisms that could improve efficacy while minimizing toxicity and providing additional beneficial effects. However, these results require further confirmation in vitro, in vivo, and in clinical studies. Overall, Bet represents a promising natural compound for complementary therapies in diabetes and metabolic disorders.

## Figures and Tables

**Table 1:** IC_50_/AC_50_ values and binding energies of Bet and metformin with key digestive, AMPK, glycolytic, and Krebs cycle enzymes. Data are presented as mean ± SD. ^α^ *p* < 0.05 Comparisons between Bet and specific inhibitors were considered statistically significant. N/A for SDH activity could not be confirmed in silico due to limited structural information regarding the active site of the enzyme.

Enzyme (PDB)	Bet		Specific Inhibitor		
	IC_50_/AC_50_ (µg/mL)	BE (kcal/mol)		IC_50_/AC_50_ (µg/mL)	BE (kcal/mol)
Lipase (1LPB)	48.8 ± 1.9	−9.3	Orlistat	62.8 ± 1.4 ^α^	−6.9
α-Amylase (5E0F)	31.9 ± 1.4	−8.9	Acarbose	37.28 ± 0.9 ^α^	−7.7
AMPK	1.83 ± 0.12	−7.9	A-769662	1.29 ± 0.04 ^α^	−10.0
HK (1V4S)	0.87 ± 0.11	−7.2		2.46 ± 0.06 ^α^	−4.6
G6PDH (6E07)	0.98 ± 0.12	−7.5		2.13 ± 0.04 ^α^	−4.6
PK (8XFD)	0.91 ± 0.03	−9.0		2.54 ± 0.03 ^α^	−5.0
IDH (5GRF)	0.76 ± 0.04	−7.8		1.98 ± 0.04 ^α^	−4.6
SDH	0.80 ± 0.03	N/A		N/A	N/A
MDH (2DFD)	0.72 ± 0.02	−7.8		2.98 ± 0.03 ^α^	−5.4
LDH (1LLD)	0.52 ± 0.03	−8.4		3.52 ± 0.05 ^α^	−4.6

## Data Availability

The original contributions presented in this study are included in the article. Further inquiries can be directed to the corresponding author.
